# Supplementary material for: Increasing quantitation in spatial single-cell metabolomics by using fluorescence as ground truth
Source: Front Mol Biosci. 2022 Nov 24;9:1021889. doi: 10.3389/fmolb.2022.1021889 (PMC9730270; doi:10.3389/fmolb.2022.1021889)
Supplement: Supplementary file 2 [file DataSheet2.docx]

**Supplemental Figures**


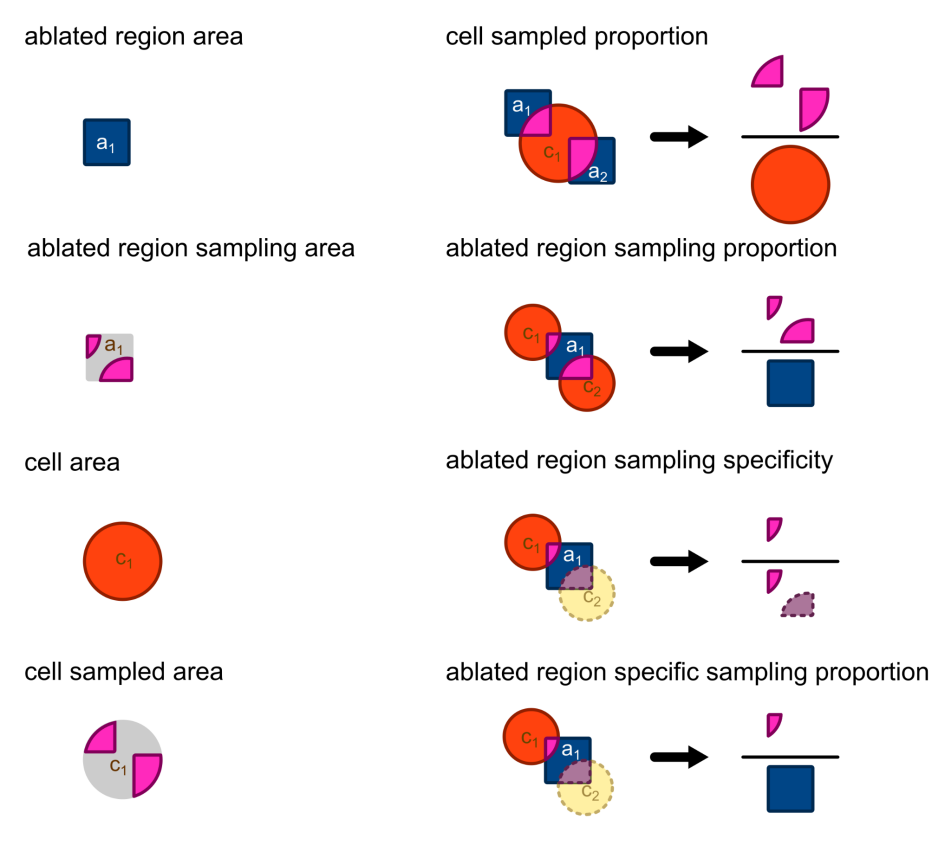


**Figure S1.** Definitions of morphological shapes and measures used in SpaceM.

**
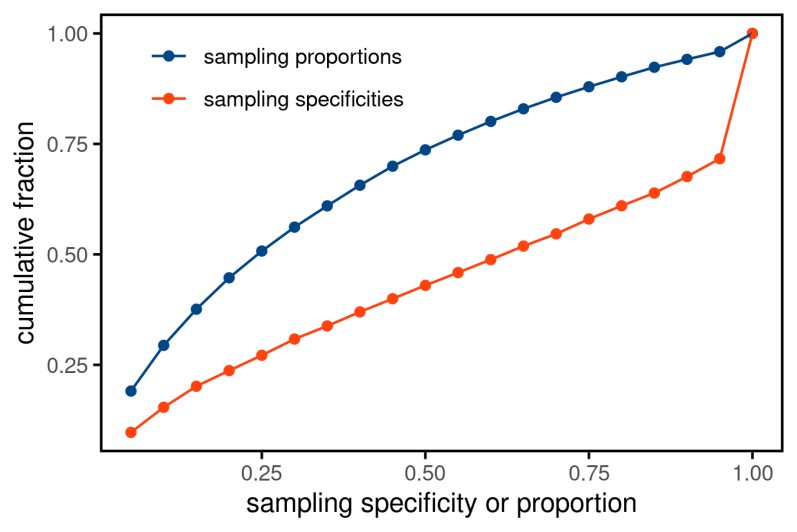
**

**Figure S2.** Cumulative fraction of sampling proportion (blue) or sampling specificity (red) of all ablated regions.


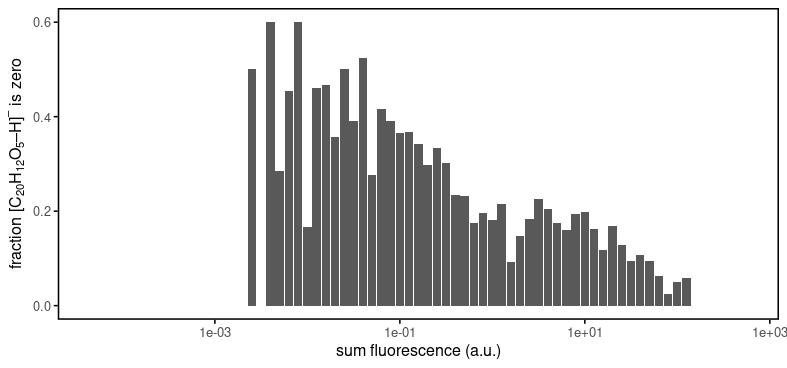


**Figure S3.** Fraction of fluorescein MS intensities for that are zero, within the same (sum fluorescein) fluorescence histogram bins as used in Figure 3B. A clear relation between increasing FDA concentration and a lower amount of drop-outs can be observed.


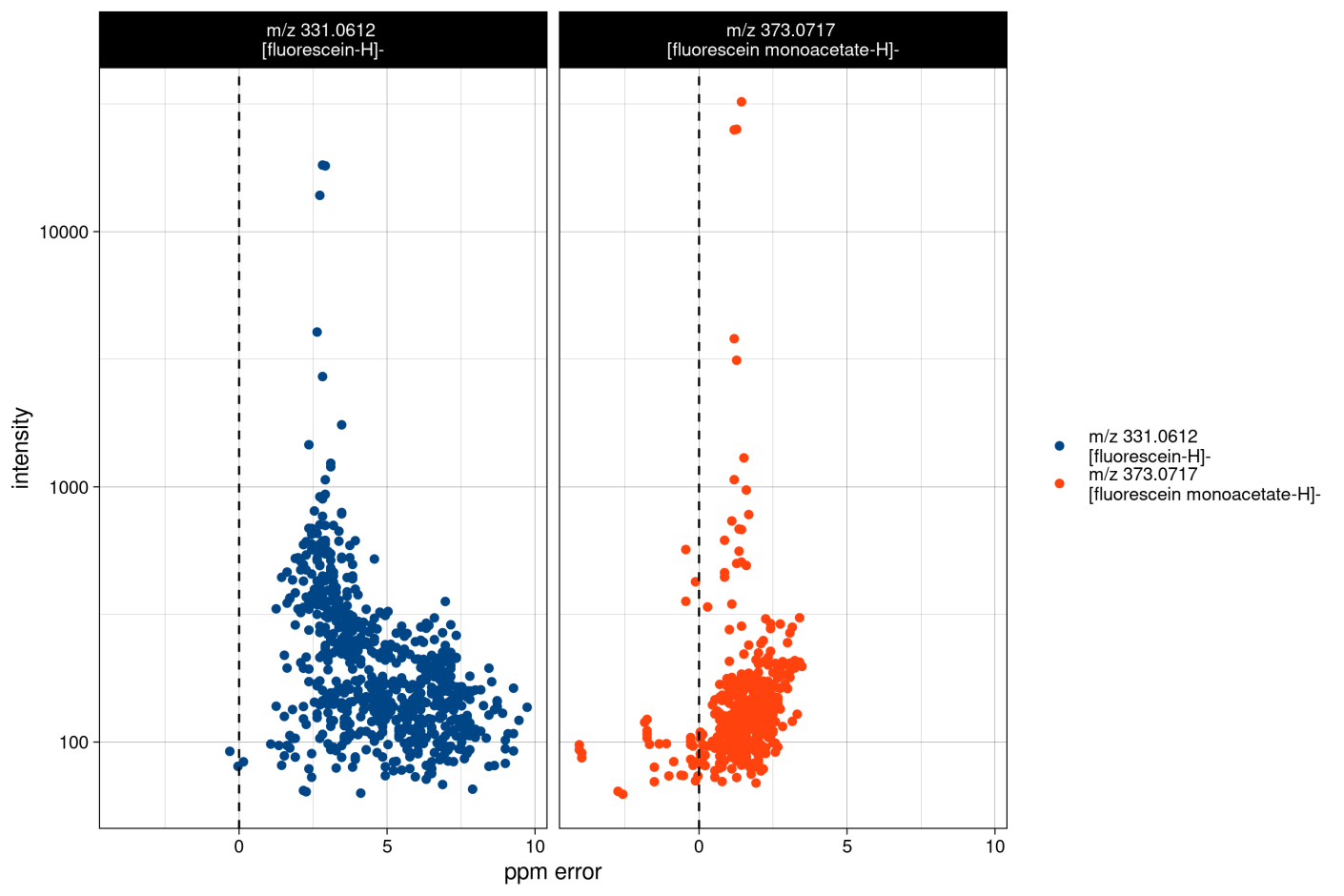


**Figure S4.** Peak intensities as a function of centroided m/z shifts in ppm of fluorescein (left) and fluorescein monoacetate (right).


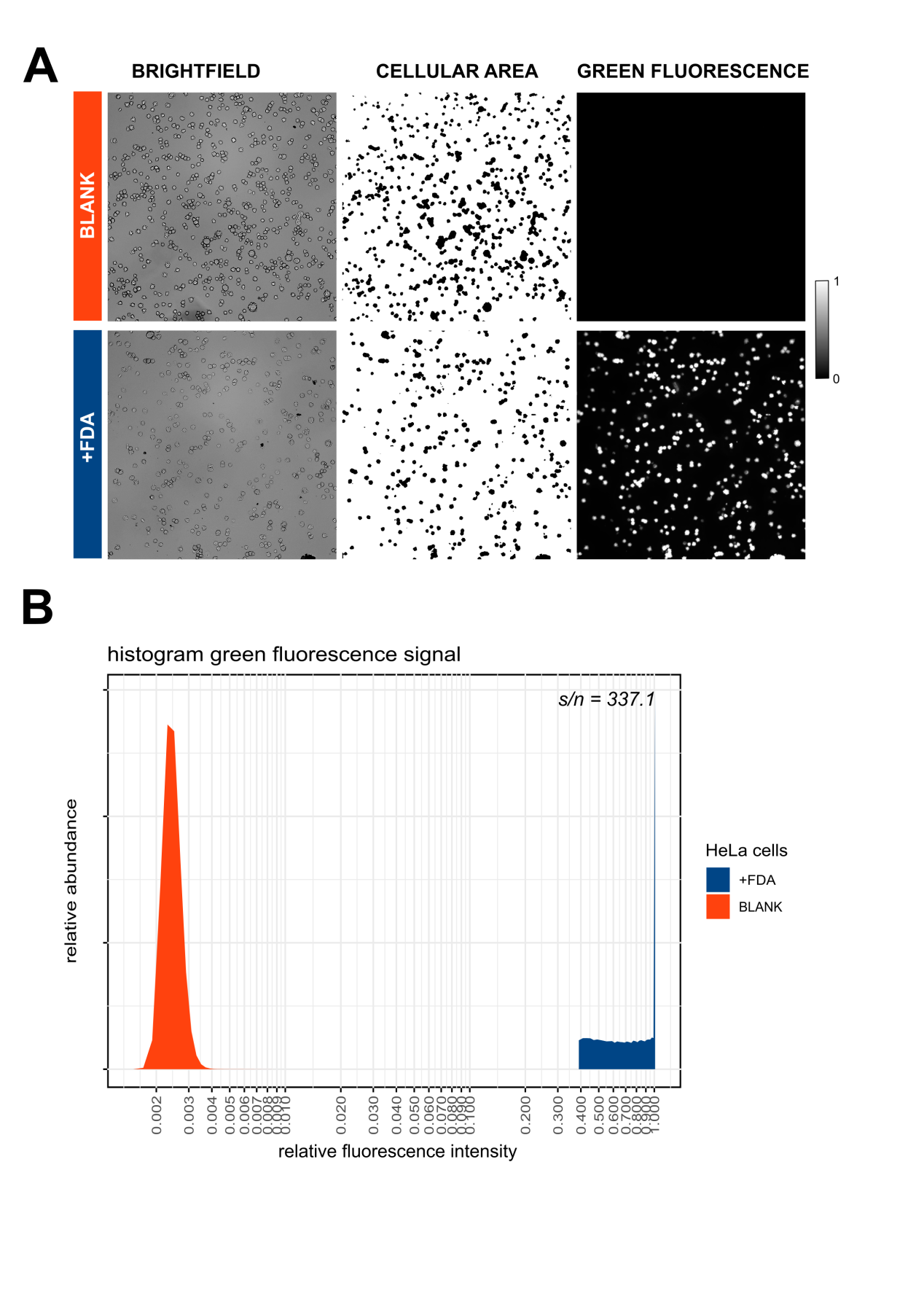


**Figure S5.** Evalulation of potential background fluorescence signals in the green channel. **A.** Brightfield and green florescence imaged of Hela cells treated with (‘FDA’) and without (‘BLANK’) 25 μM FDA. The middle panel shows the celluar areas in black. Fluorescent signals in the blank were negligible. **B.** Histograms of the fluorescence intensity in the cellular areas of the same conditions. The x-axis is shown in log-scale, the signal-to-noise levels (S/N) are shown in right-top corner of the panel.


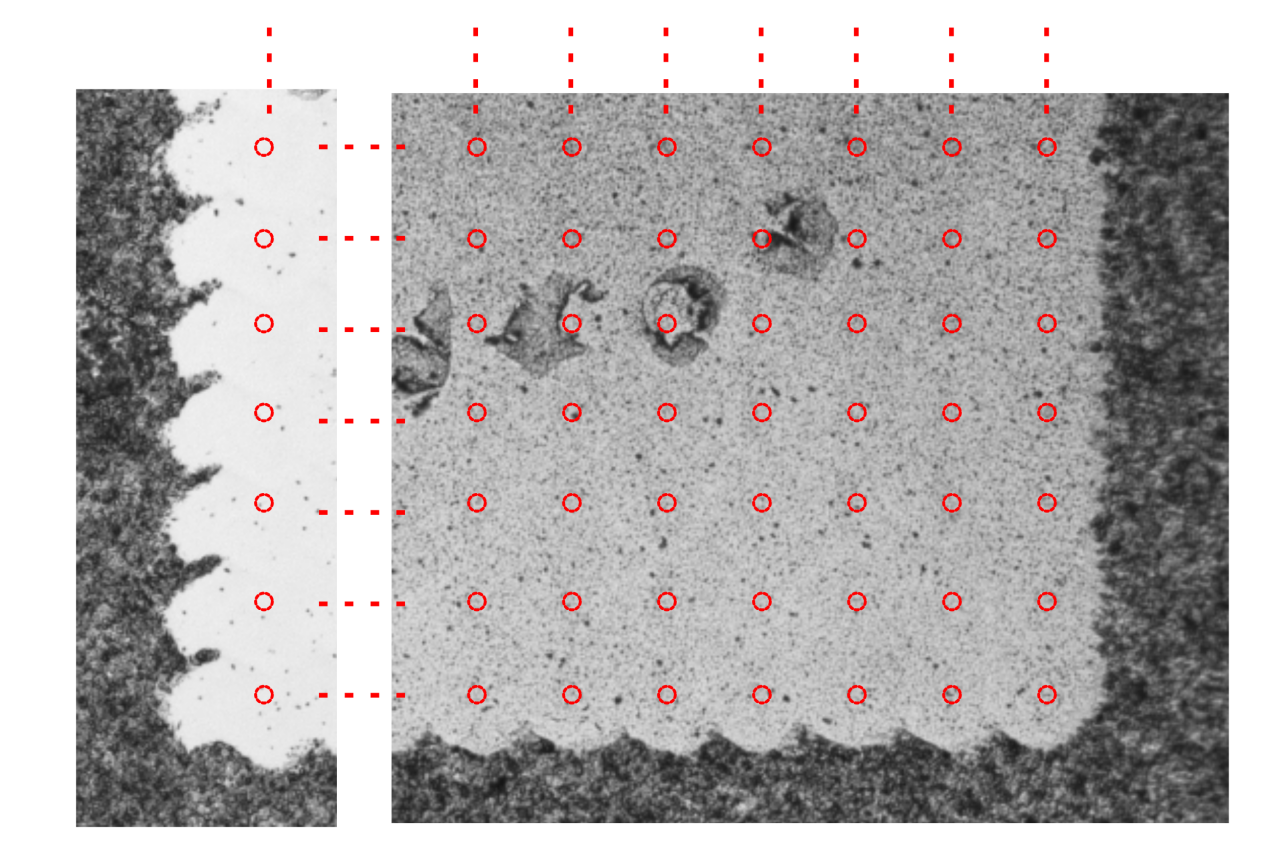


**Figure S6.** Example brightfield-image of the post-MALDI acquisition showing the laser-ablated regions. The red circles show the centers of the ablated regions.
